# Socioeconomic disparities in risk of financial toxicity following elective cardiac operations in the United States

**DOI:** 10.1371/journal.pone.0292210

**Published:** 2024-01-31

**Authors:** Alberto Romo Valenzuela, Nikhil L. Chervu, Yvonne Roca, Yas Sanaiha, Saad Mallick, Peyman Benharash

**Affiliations:** 1 Cardiovascular Outcomes Research Laboratories (CORELAB), Division of Cardiac Surgery, Department of Surgery, David Geffen School of Medicine, University of California, Los Angeles, California, United States of America; 2 Department of Surgery, David Geffen School of Medicine, University of California, Los Angeles, California, United States of America; University of Louisville, UNITED STATES

## Abstract

**Background:**

While insurance reimbursements allay a portion of costs associated with cardiac operations, uncovered and additional fees are absorbed by patients. An examination of financial toxicity (FT), defined as the burden of patient medical expenses on quality of life, is warranted. Therefore, the present study used a nationally representative database to demonstrate the association between insurance status and risk of financial toxicity (FT) among patients undergoing major cardiac operations.

**Methods:**

Adults admitted for elective coronary artery bypass grafting (CABG) and isolated or concomitant valve operations were assessed using the 2016–2019 National Inpatient Sample. FT risk was defined as out-of-pocket expenditure >40% of post-subsistence income. Regression models were developed to determine factors associated with FT risk in insured and uninsured populations. To demonstrate the association between insurance status and risk of FT among patients undergoing major cardiac operations.

**Results:**

Of an estimated 567,865 patients, 15.6% were at risk of FT. A greater proportion of uninsured patients were at risk of FT (81.3 vs. 14.8%, *p*<0.001), compared to insured. After adjustment, FT risk among insured patients was not affected by non-income factors. However, Hispanic race (Adjusted Odds Ratio [AOR] 1.60), length of stay (AOR 1.17/day), and combined CABG-valve operations (AOR 2.31, all *p*<0.05) were associated with increased risk of FT in the uninsured.

**Conclusion:**

Uninsured patients demonstrated higher FT risk after undergoing major cardiac operation. Hispanic race, longer lengths of stay, and combined CABG-valve operations were independently associated with increased risk of FT amongst the uninsured. Conversely, non-income factors did not impact FT risk in the insured cohort. Culturally-informed reimbursement strategies are necessary to reduce disparities in already financially disadvantaged populations.

## Introduction

Coronary artery bypass grafting (CABG) and valve operations are associated with an average of $40,000 in inpatient costs and are estimated to result in nearly $7 billion in total charges across the US, each year [1]. Although these operations are generally reimbursed by insurance plans, uncovered and additional costs due to complications may need to be absorbed by patients and individual institutions [2–4]. With US healthcare expenditures rising by 4–7% per year, uninsured patients are at particularly high risk of financial liability [5]. Moreover, prior work has demonstrated uninsured patients to have higher rates of complications, further increasing costs [6].

The term “financial toxicity” (FT) refers to the negative impact of medical expenses on patients’ financial well-being, quality of life, and optimal receipt of healthcare [7, 8]. Although studied in the context of cancer and trauma, implications of FT in cardiac surgery remain unexplored [9–14]. However, research in medically managed cardiovascular diseases has found lack of insurance to increase the risk of FT [15, 16]. In particular, cardiac surgery patients continue to have significant outpatient costs, often requiring continued medical therapy and rehabilitation services. A thorough examination of outcomes, costs, and subsequent risk of FT after major cardiac operations is thus warranted.

The present study evaluated the relationship of insurance status with the risk of financial toxicity following elective major cardiac operations. We hypothesize lack of insurance to be associated with increased FT risk. Furthermore, we postulate the presence of racial disparities among uninsured patients with higher risk of FT. Finally, we assessed short-term outcomes stratified by insurance status, positing that absence of insurance would be linked to increased rates of major adverse events and resource utilization.

## Materials and methods

**Fig 1:**
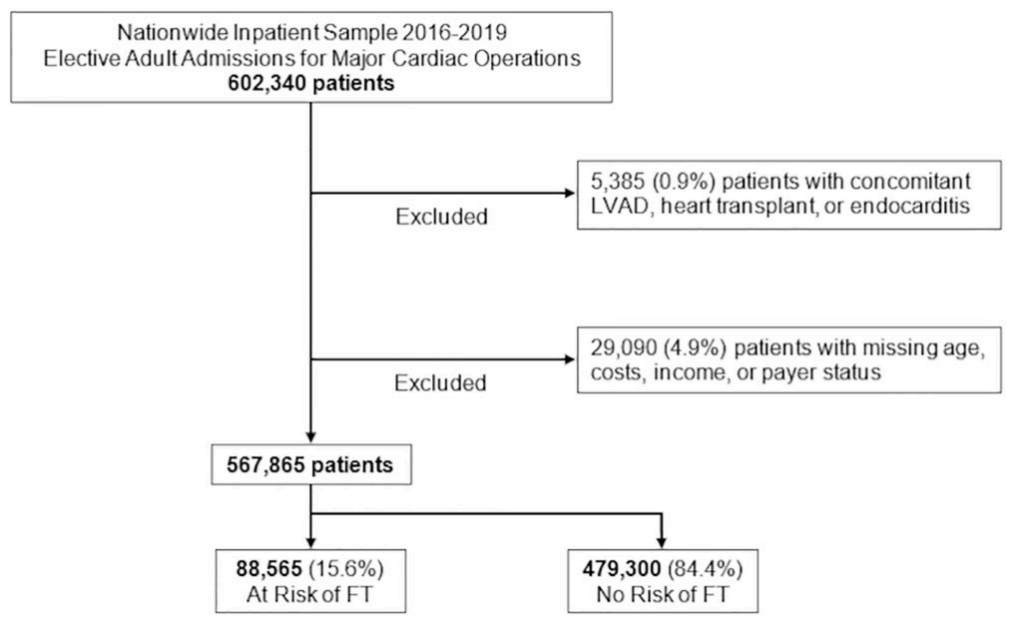
Exclusion criteria; LVAD, left ventricular assist device.

This project was deemed exempt from full review by the Institutional Review Board (IRB) at the University of California, Los Angeles s (IRB# 17–001112). Patient consent (written and oral) was waived due to the de-identified nature of the National Inpatient Sample database. The 2016 to 2019 National Inpatient Sample (NIS) was used to identify all adult (≥18 years) elective admissions for coronary artery bypass grafting (CABG) and isolated or concomitant valve operations using appropriate *International Classification of Disease*, *Tenth Revision* (ICD-10) codes (S1 Table). Administered by the Healthcare Cost and Utilization Project (HCUP), the NIS is the largest all-payer inpatient database and provides accurate estimates for roughly 97% of all U.S. hospitalizations [17]. Admissions with concomitant left ventricular assist device placement, heart transplant, and endocarditis were excluded from analysis. Records missing values for age, sex, costs, income, or primary payer status, were further excluded (4.9%; Fig 1).

Additional patient and hospital characteristics were defined using the NIS data dictionary and included age, sex, race, income quartile, teaching status, bed size, and hospital geographic region [17]. Insurance status was identified according to existing HCUP definitions. Self-pay patients were categorized as *Uninsured*, while patients with private, government-sponsored insurance, or other types of coverage were considered *Insured*. The van Walraven modification of the Elixhauser Comorbidity Index was used to quantify the burden of chronic conditions [18, 19]. Hospitalization costs were obtained via application of center-specific cost-to-charge ratios to overall charges with inflation adjustment to the 2019 Personal Health Index [20, 21].

The primary endpoint of the study was risk of FT. Secondary endpoints included major adverse events (MAE), length of stay (LOS), hospitalization costs, and non-home discharge. MAE was a composite of in-hospital mortality and perioperative complications derived from current Society of Thoracic Surgeons (STS) quality metrics: stroke or transient ischemic attack (TIA), prolonged ventilation >96 hours, acute renal failure requiring dialysis, and reoperation [22].

Risk of FT was calculated by adapting previously used methods in the oncologic and trauma literature [11, 13]. Gamma distribution probability density functions were first constructed to determine individual patient incomes [23]. The use of gamma distribution-based models to estimate patient income has been well-described in both the biostatistical and epidemiological fields [24, 25]. Next, shape and scale parameters were derived from NIS ZIP code-based income quartiles which were supplemented with data from the US Census Bureau [17, 26]. The shape parameter was determined to be 1.568 based on a GINI coefficient of 0.415 and prior work by Shrime et al. [27, 28]. The NIS-provided variable for income quartiles (ZIPINC_QRTL) sets specific ranges for each year. Due to inflation and other factors, this value changes for each year. To better model overall income distribution throughout the study period, we took the mean of the lowest three income quartiles for each of the four years studied. For the top quartile, however, only the bottom of the range is provided. Therefore, the lower limit of the ninth decile was used for the highest income quartile (S2 Table) [29]. These incomes were divided by the shape parameter to obtain the scale parameters. Next, post-subsistence income was derived using food and maximum out-of-pocket expenditure (OOP) data from the Centers for Medicare and Medicaid Services [29–33]. Maximum OOP was attained via in-network essential health benefit payments for individual healthcare plans in 2019. Finally, risk of FT was assessed by using mean maximum OOP expenditures or hospitalization costs that resulted in greater than 40% of the post-subsistence income for insured and uninsured patients, respectively [10, 11].

### Statistical analysis

Categorical variables are reported as percentages (%) while continuous variables are shown as means with standard deviation (SD) or medians with interquartile range (IQR). The Adjusted Wald and Pearson’s χ^2^ tests were used to determine the significance of intergroup differences for continuous and categorical variables, respectively. Cuzick’s non-parametric rank test was used to assess the significance of temporal trends (*nptrend*) [34]. Multivariable regression models used to determine patient and hospital factors associated with risk of FT. Separate models were constructed for insured and uninsured patients. Additional regression models were then developed to assess the association of insurance status with the secondary outcomes. Covariate selection for regression models was guided by the Least Absolute Shrinkage Selection Operator (LASSO). This algorithm increases prediction accuracy while reducing collinearity and model overfitting [35]. Covariates provided for this algorithm included insurance status, type of operation, patient age, patient sex, patient race, weekend admission status, year of admission, income quartile, hospital setting, hospital region, Elixhauser Comorbidity Index, and specific comorbid conditions (neurological disorder, cardiac arrhythmia, congestive heart failure, pulmonary circulatory disorder, peripheral vascular disease, hypertension, chronic lung disease, end-stage renal disease, chronic liver disease, coagulopathy, obesity, weight loss prior to surgery, diabetes, and rheumatologic disorder). All models were optimized via the area under the receiver-operating characteristic curve (C-statistic) as well as Akaike and Bayesian information criteria [36]. Regression outputs are reported as adjusted odds ratios (AOR) or beta coefficients (β) with 95% confidence intervals (CI). A *p*-value < 0.05 was considered statistically significant. All statistical analyses were completed using Stata 16 (StataCorp, College Station, TX).

## Results

### Estimated income and mean maximum out-of-pocket expenditure

To calculate the risk of FT, gamma distributions were used to estimate patient income by quartile (S1 Fig). Food expenses were then obtained from the Bureau of Labor and Statistics, which ranged from $3,700 to $17,100 [26, 28–30]. Next, post-subsistence income was calculated for the different types of insurance. The median post-subsistence income for uninsured patients was $41,600 [19,700–82,200], $46,300 [22,300–87,200] for Medicare patients, $41,500 [20,100–78,300] for Medicaid patients, and $48,800 [23,700–90,400] for privately insured patients. The mean maximum OOP expenditure was $5,000 (range 0–7,900), which is similar to in-network numbers reported by the 2019 United Benefits Advisors Health Plan Survey [21]. Furthermore, the maximum limit for a US health plan that covered an individual in 2019 under the Affordable Care Act was $7,900 [37].

### Risk of financial toxicity

**Fig 2:**
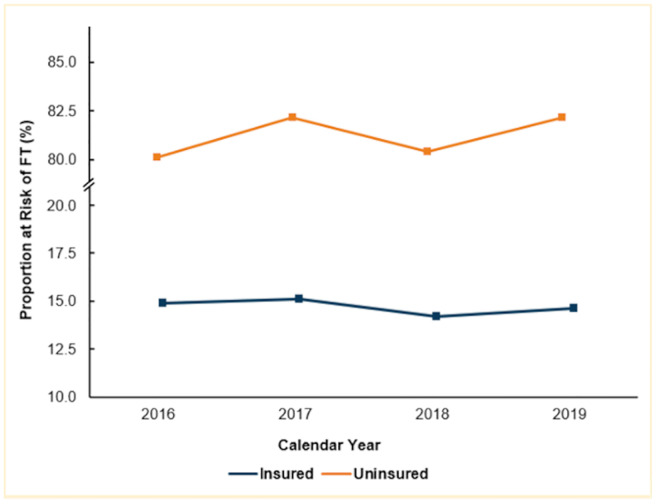
Temporal trends of risk of financial toxicity (FT) between insured and uninsured patients, 2016–2019; no significant trends noted.

Of an estimated 567,865 patients, 15.6% of patients were at risk of FT. Notably, a higher proportion of uninsured patients were at increased risk of FT (81.3 vs 14.8%, *p*<0.001) compared to their insured counterparts. FT risk remained steady during the four-year study period for both insured (*nptrend* = 0.67) and uninsured patients (*nptrend* = 0.24; Fig 2). Notably, *Uninsured* was consistently at greater risk of FT compared to the Insured group across all four years. Compared to those not at risk of FT, those at risk of FT were younger (65.0 ± 11.4 vs 65.9 ± 11.1, *p*<0.001) and more commonly female (30.4 vs 23.4%, *p* = 0.007), but had similar Elixhauser Comorbidity Indices (5 [3–6] vs 4 [3–6], *p* = 0.15). Those at risk of FT were more commonly of Black (7.3 vs 5.4%) and Hispanic (7.3 vs 6.0%, both *p*<0.001) race, compared to others. Additionally, patients at risk of FT were most likely to be classified in the lowest income quartile (37.5 vs 21.5%, *p*<0.001), compared to those not at risk of FT. Those at risk of FT were more frequently treated within the Southern NIS geographic region (43.6 vs 36.5%, *p*<0.001) and more likely managed at urban, non-teaching institutions (14.1 vs 13.6%, *p*<0.001). Finally, patients at risk of FT were more likely to undergo isolated CABG (53.6 vs 52.2%, *p* = 0.004; Table 1).

**Table 1:** Baseline patient, clinical, and hospital characteristics of patients undergoing major elective cardiac surgery stratified by risk of financial toxicity (FT).

	At Risk of FT (n = 88,565)	No Risk of FT (n = 479,300)	p-value
Age (years, mean ± SD)	65.0 ± 11.4	65.9 ± 11.1	<0.001
Female (%)	26,905 (30.4)	140,765 (23.4)	0.007
Elixhauser Comorbidity Index (median, IQR)	5 [3 – 6]	4 [3 – 6]	0.15
*Race (%)*			<0.001
White	68,225 (77.0)	461,275 (79.3)	
Black	6,435 (7.3)	25,955 (5.4)	
Hispanic	6,450 (7.3)	28,660 (6.0)	
Asian/Pacific Islander	1,905 (2.2)	13,855 (2.9)	
Other	2,350 (2.7)	13,155 (2.7)	
*Income quartile (%)*			<0.001
0-25th	33,210 (37.5)	102,450 (21.5)	
26th-50th	27,350 (30.9)	122,830 (25.6)	
51st-75th	18,360 (20.7)	129,890 (27.1)	
76th-100th	9,645 (10.9)	124,130 (25.9)	
*Major Cardiac Surgeries (%)*			0.004
Isolated CABG	47,505 (53.6)	250,420 (52.2)	
Single Valve	26,115 (29.5)	147,385 (30.8)	
CABG + Valve	10,990 (12.4)	60,475 (12.6)	
Multiple Valves	3,955 (4.5)	21,015 (4.4)	
*Primary payer (%)*			<0.001
Private	27,160 (30.7)	162,335 (33.9)	
Medicare	48,105 (54.3)	275,885 (57.6)	
Medicaid	5,375 (6.1)	28,100 (5.9)	
Other payer	2,090 (2.4)	11,635 (2.4)	
Uninsured	5,835 (6.6)	1,345 (0.3)	
*Hospital region (%)*			<0.001
Northeast	12,630 (14.3)	88,019 (18.4)	
Midwest	23,410 (26.4)	117,950 (19.3)	
South	38,625 (43.6)	175,000 (36.5)	
West	13,900 (15.7)	89,540 (18.7)	
*Hospital teaching status (%)*			<0.001
Urban teaching	73,380 (82.9)	404,470 (84.4)	
Urban non-teaching	12,500 (14.1)	65,225 (13.6)	
Rural	2,685 (3.0)	9,605 (2.0)	
*Bed size (%)*			0.79
Large	58,105 (65.6)	313,250 (65.4)	
Medium	21,175 (23.9)	115,920 (24.2)	
Small	9,285 (10.5)	50,130 (10.5)	
*Comorbidities (%)*			
Cardiac arrhythmia	47,015 (53.1)	260,325 (54.3)	0.002
Coagulopathy	24,650 (27.8)	138,625 (28.9)	0.004
Chronic liver disease	2,905 (3.3)	14,780 (3.1)	0.17
Chronic lung disease	19,160 (21.6)	96,185 (20.1)	<0.001
Congestive heart failure	21,755 (24.6)	113,405 (23.7)	0.013
Diabetes	33,930 (38.3)	177,185 (37.0)	<0.001
End-stage renal disease	15,405 (17.4)	84,720 (17.7)	0.37
Hypertension	73,815 (83.3)	397,750 (83.0)	0.26
Neurologic disease	5,050 (5.7)	25,805 (5.4)	0.086
Obesity	24,615 (27.8)	126,785 (26.5)	<0.001
Peripheral vascular disease	15,610 (17.6)	87,130 (18.2)	0.078
Pulmonary circulatory disorder	8,800 (9.9)	44,725 (9.3)	0.015
Rheumatologic disorder	2,350 (2.7)	13,080 (2.7)	0.56
Weight loss	2,315 (2.6)	11,815 (2.5)	0.25

SD, standard deviation; IQR, interquartile range; CABG, coronary artery bypass grafting

**Fig 3:**
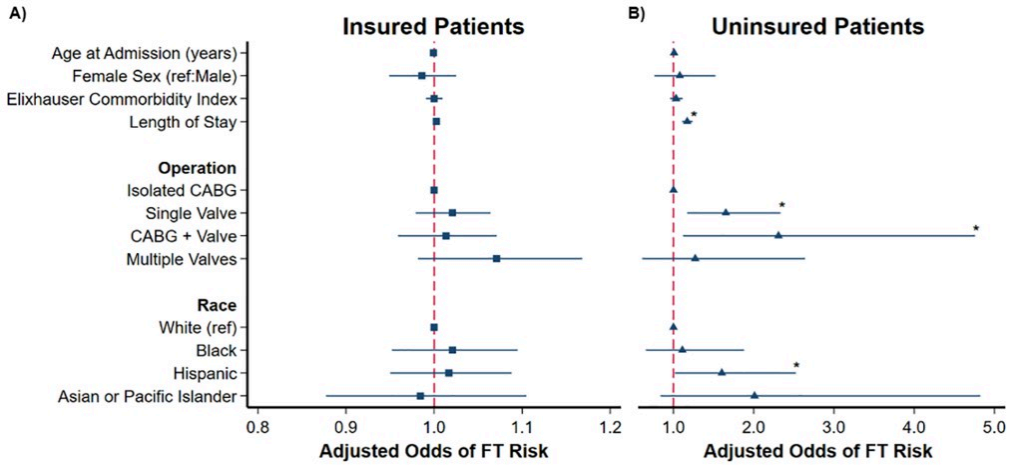
Patient and hospital characteristics associated with risk of financial toxicity (FT) among (A) insured and (B) uninsured patients; Insured C-statistic: 0.64, Uninsured C-statistic 0.75; adjusted odds ratios are shown with 95% confidence intervals; CABG, coronary artery bypass grafting; *p<0.05.

After adjustment, decreasing income quartile was the only tabulated characteristic associated with increased odds of FT risk in insured patients. Conversely, increased LOS (AOR 1.17, 95% CI 1.11–1.24, *p*<0.001) and Hispanic race (AOR 1.60, 95% CI 1.02–2.52; ref: White) had higher adjusted odds of FT risk in the uninsured. Finally, single valve (AOR 1.65, 95% CI 1.17–2.33, *p* = 0.004) and combined CABG and valve operations (AOR 2.31, 95% CI 1.12–4.76, *p* = 0.02) were associated with increased odds of FT risk in uninsured patients, compared to isolated CABG (Fig 3).

### Impact of insurance status on outcomes

7,180 (1.3%) of the study cohort were noted to be uninsured. Amongst the insured population, 357,465 (63.8% of *Insured*) had government-funded insurance with the rest being privately or otherwise insured. Compared to *Insured*, *Uninsured* was younger (56.3 ± 10.9 vs 65.9 ± 11.1 years, *p*<0.001) and had lower Elixhauser Indices (4 [3–6] vs 5 [3–6], *p*<0.001). They were, however, comparable in female composition (29.0 vs 29.5, *p* = 0.65). The *Uninsured* cohort were more commonly Black (9.5 vs 5.7%) and Hispanic (14.9 vs 6.1%, both *p*<0.001), compared to others. In addition, *Uninsured* patients were more likely to be in the lowest income quartile (35.6 vs 23.7%, *p*<0.001) compared to their *Insured* counterparts. *Uninsured* patients were more commonly treated within the Southern NIS geographic region (63.2 vs 37.3%, *p*<0.001), but were equally managed at urban teaching (82.5 vs 84.2%, *p* = 0.36) hospitals. Finally, *Uninsured* patients more frequently underwent isolated CABG (58.0 vs 52.8%, *p*<0.001) and less commonly combined CABG and valve operations (7.9 vs 12.6%, *p*<0.001; Table 2). On unadjusted analysis, the *Uninsured* cohort had similar rates of MAE (17.2 vs 18.7%, *p* = 0.15). Despite having longer LOS (7 [5–9] vs 6 [5–9], *p* = 0.003), both *Uninsured* and *Insured* patients had similar hospitalization costs (39.8 [30.9–53.0] vs 39.9 [31.0–53.8], *p* = 0.04). *Uninsured* patients additionally had lower rates of non-home discharge (7.6 vs 20.7%, *p*<0.001; Table 3).

**Table 2:** Baseline patient, clinical, and hospital characteristics of patients undergoing major elective cardiac surgery stratified by insurance status.

	Insured (n = 560,685)	Uninsured (n = 7,180)	p-value
Age (years, mean ± SD)	65.9 ± 11.1	56.3 ± 10.9	<0.001
Female (%)	165,590 (29.5)	2,080 (29.0)	0.65
Elixhauser Comorbidity Index (median, IQR)	5 [3 – 6]	4 [3 – 6]	<0.001
*Race (%)*			<0.001
White	443,165 (79.0)	4,760 (66.3)	
Black	31,705 (5.7)	685 (9.5)	
Hispanic	34,040 (6.1)	1,070 (14.9)	
Asian/Pacific Islander	15,545 (2.8)	215 (3.0)	
Other	15,155 (2.7)	350 (4.9)	
*Income quartile (%)*			<0.001
0-25th	133,105 (23.7)	2,555 (35.6)	
26th-50th	148,140 (26.4)	2,040 (28.4)	
51st-75th	146,640 (26.2)	1,610 (22.4)	
76th-100th	132,800 (23.7)	975 (13.6)	
*Major Cardiac Surgeries (%)*			<0.001
Isolated CABG	293,835 (52.4)	4,090 (58.0)	
Single Valve	171,325 (30.6)	2,175 (30.3)	
CABG + Valve	70,900 (12.6)	565 (7.9)	
Multiple Valves	24,620 (4.4)	350 (4.9)	
*Hospital region (%)*			<0.001
Northeast	100,130 (17.9)	520 (7.2)	
Midwest	148,731 (26.5)	1,420 (19.8)	
South	209,090 (37.3)	4,535 (63.2)	
West	102,735 (18.3)	705 (9.8)	
*Hospital teaching status (%)*			0.36
Urban teaching	471,925 (84.2)	5,925 (82.5)	
Urban nonteaching	76,656 (13.7)	1,070 (14.9)	
Rural	12,105 (2.2)	185 (2.6)	
*Bed size (%)*			0.52
Large	366,615 (65.4)	4,740 (66.0)	
Medium	135,315 (24.1)	1,780 (24.8)	
Small	58,755 (10.5)	660 (9.2)	
*Comorbidities (%)*			
Cardiac arrhythmia	304,175 (54.3)	3,165 (44.1)	<0.001
Coagulopathy	161,410 (28.8)	1,865 (26.0)	0.034
Chronic liver disease	17,505 (3.1)	180 (2.5)	0.19
Chronic lung disease	114,035 (20.3)	1310 (18.2)	0.071
Congestive heart failure	133,075 (23.7)	2,085 (29.0)	<0.001
Diabetes	208,450 (37.2)	2,665 (37.1)	0.97
End-stage renal disease	99,225 (17.7)	900 (12.5)	<0.001
Hypertension	465,770 (83.1)	5,795 (80.7)	0.018
Neurologic disease	30,480 (5.4)	375 (5.2)	0.73
Obesity	149,525 (26.7)	1,875 (26.1)	0.66
Peripheral vascular disease	101,635 (18.1)	1,105 (15.4)	0.0008
Pulmonary circulatory disorder	52,675 (9.4)	850 (11.8)	0.002
Rheumatologic disorder	15,345 (2.7)	85 (1.2)	<0.001
Weight loss	13,915 (2.5)	215 (3.0)	0.21

SD, standard deviation; IQR, interquartile range; CABG, coronary artery bypass grafting

**Table 3:** Unadjusted outcomes of patients undergoing major elective cardiac surgery stratified by insurance status.

	Insured (n = 560,685)	Uninsured (n = 7,180)	p-value
Major Adverse Events (%)	104,980 (18.7)	1,235 (17.2)	0.15
**Individual Complications (%)**			
Mortality	9,670 (1.7)	110 (1.5)	0.59
Stroke/TIA	9,910 (1.8)	125 (1.7)	0.94
Prolonged Ventilation	11,690 (2.1)	160 (2.2)	0.71
Acute Renal Failure	87,730 (15.6)	1,055 (14.7)	0.33
Reoperation	6,945 (1.2)	100 (1.4)	0.59
**Resource Utilization**			
LOS (days, median, IQR)	6 [5 – 8]	7 [5 – 9]	0.003
Costs ($1000s, median, IQR)	39.9 [31.0–53.8]	39.8 [30.9–53.0]	0.48
Non-Home Discharge (%)	116,060 (20.7)	545 (7.6)	<0.001

TIA, transient ischemic attack; IQR, interquartile range

After risk-adjustment, lack of insurance was not significantly associated with altered odds of MAE (AOR 1.15, 95% CI 0.98–1.34, *p* = 0.09). However, age (AOR 1.02, 95% CI 1.02–1.02, *p*<0.001), combined CABG and valvular operations (AOR 1.08, 95% CI 1.02–1.14, *p* = 0.008), and Black (AOR 1.37, 95% CI 1.27–1.48, *p*<0.001) or Hispanic (AOR 1.15, 95% CI 1.06–1.24, *p* = 0.001) races were among factors associated with increased adjusted odds of MAE. Female sex (AOR 0.84, 95% CI 0.81–0.96, *p*<0.001) and isolated valvular operations (AOR 0.70, 95% CI 0.67–0.74, *p*<0.001) were conversely associated with decreased odds of MAE. Compared to the insured cohort, uninsured had significantly longer adjusted LOS (β 1.07, 95% CI 1.03–1.10, *p*<0.001) but similar hospitalization costs (β -500, 95% CI -1700, +800, *p* = 0.47). Finally, uninsured patients had lower adjusted odds of non-home discharge (AOR 0.46, 95% CI 0.35–0.60, *p*<0.001; Table 4).

**Table 4:** Adjusted outcomes of *Uninsured* patients undergoing major elective cardiac surgery, outputs reported as adjusted odds ratios (AOR) or β coefficients.

	AOR or β Coefficient	95% CI	p-value
Major Adverse Events (AOR)	1.15	0.98, 1.34	0.09
**Individual Complications (AOR)**			
Mortality	1.03	0.62, 1.72	0.90
Stroke/TIA	1.09	0.70, 1.68	0.71
Prolonged Ventilation	1.07	0.71, 1.60	0.74
Acute Renal Failure	1.23	1.04, 1.45	0.02
Reoperation	1.25	0.80, 1.93	0.32
**Resource Utilization**			
LOS (β, days)	+1.07	+1.03, +1.10	<0.001
Costs (β, $1000s)	-0.45	-1.68, +0.78	0.47
Non-Home Discharge (AOR)	0.46	0.35, 0.60	<0.001

CI, confidence interval; TIA, transient ischemic attack; LOS, length of stay

## Discussion

Using a nationwide all-payer database, the current study presents the first analysis of FT risk among patients undergoing major elective cardiac operations. We additionally compared short-term outcomes between insured and uninsured patients. Notably, although FT risk remained stable throughout the study period, uninsured patients were more than five times as likely to be at risk of FT. In addition, while no demographic or clinical factors were associated with increased risk of FT amongst insured patients, Hispanic race, and increased LOS were among factors linked with increased risk of FT in the uninsured. Finally, uninsured patients had longer adjusted LOS and decreased odds of non-home discharge despite similar clinical outcomes.

Despite only accounting for 1.3% of the total study population, 81.3% of uninsured patients were at risk of FT compared to 14.8% of insured patients. Our results mirror those provided by Khera et al., who noted uninsured patients with atherosclerotic cardiovascular disease to have a two-fold increase in odds of financial hardship [16]. Patients with cardiovascular disease require frequent medical and prescription medications. Unfortunately, FT leads to higher rates of medication non-adherence and delayed medical care as a result of financial strain [7, 16]. Consequently, quality of life may be particularly impacted in cardiac surgery patients experiencing FT. Contrary to prior work, however, risk of FT remained consistent throughout the study period [11, 13]. Differences in baseline patient demographics in our study may explain this disparity. While trauma patients are frequently young and otherwise healthy, those requiring cardiac surgery have a higher burden of comorbid disease [12, 13, 38]. Similarly, although cancer patients ultimately require significant care, many are healthy prior to their diagnosis. Specifically, while 97.4% of our cohort had any comorbid disease, a 2020 study found that only two-thirds of cancer patients had a chronic condition [39]. Risk of FT in cardiac surgery patients, therefore, is likely associated with the increased, one-time costs of surgical care as opposed to a significant change from healthy to unhealthy status. Regardless of trends, the persistent disparity in FT risk between insured and uninsured patients highlights the need for increased insurance coverage in the broader patient population.

Interestingly, while risk of FT was not impacted by racial or hospital factors among the insured, uninsured patients were affected by non-income factors. Hispanic race, valvular operations, and LOS were specifically associated with increased risk of FT in the uninsured. Hispanic populations experience higher rates of cardiovascular disease, diabetes, and renal disease, compared to their White counterparts [40]. More importantly, Hispanic patients have higher rates of under insurance [40, 41]. This has been attributed to language barriers, lower rates of post-high school education, decreased health literacy, and cultural or immigration barriers [42, 43]. With lower incomes and increased costs due to higher rates of chronic conditions, our results are not unexpected. Uninsured patients undergoing valvular operations, specifically single valve and CABG + valve, likewise had higher odds of FT risk. Research by our group and others has demonstrated both isolated valve and combined CABG + valve operations to be costlier compared to isolated CABG [44, 45]. Surprisingly, multivalve operations were not similarly associated with increased risk of FT. This is likely due to its overall rarity, with fewer than 5% of operations in our cohort classified as multivalve surgery. Finally, increased LOS was associated with increased risk of FT in uninsured patients. Given these factors, our data suggests that increasing overall insurance coverage is necessary in reducing FT risk in cardiac surgery patients. Moreover, strategies addressing health literacy, employment and wage inequality, and transportation may mitigate FT disparity in uninsured, Hispanic patients.

Upon further analysis, the present study did not find any significant differences in MAE between insured and uninsured patients. These findings are congruent with prior literature examining major operations and CABG [46, 47]. Uninsured status has been linked with worse outcomes in emergent operations and medically managed conditions [48–50]. However, our study cohort is limited to patients undergoing elective operations, requiring significant preoperative workup and risk stratification. As a result, differences in clinical outcomes between insured and uninsured patients may be minimized by patient selection. Our data additionally showed uninsured patients to have longer adjusted LOS and decreased odds of non-home discharge, similar to prior studies [46]. This may be due to inadequate post-discharge rehabilitation enrollment or difficulties in discharge placement for uninsured patients. Data from the American Heart Association has shown lack of insurance coverage or access to cardiac rehabilitation to be the biggest contributors to reduced utilization [51]. Without adequate access to post-hospitalization resources or inpatient facilities, patients may need to achieve higher thresholds to ensure safe home discharge. Although current standardized procedures may mitigate outcome disparities, expansion of rehabilitation and care coordination is imperative in reducing differences in inpatient resource utilization.

The present study has several important limitations, particularly those that are inherent to the use of administrative data. The NIS relies on accurate coding and is therefore subject to variability in coding practices that is mainly used for insurance reimbursement. Therefore, it possesses limited granular data, and thus does not provide patient data on vitals, lab values, or information regarding intensive care utilization. Our exclusion of non-elective admissions may increase the relative proportion of higher income and educated patients. However, due to the lack of lab data, vitals, EKG results, and other preoperative interventions, incorporation of these patients may have otherwise increased the heterogeneity of the patient population. Additionally, both overall costs and patient income are estimated. Specifically, costs are calculated from inpatient charges alone, with costs due to outpatient care, rehabilitation, or home health services not available for analysis. Income was likewise derived from patient zip code. Lastly, due to the observational nature of the study, we cannot establish causal relationships.

## Conclusion

In summary, uninsured patients were more likely to be at risk of FT throughout the study period. Furthermore, racial minority status, longer LOS, and valvular operations were independently associated with increased FT risk in uninsured patients. These disparities were not noted in insured patients, who were unaffected by non-income clinical or demographic factors. Finally, lack of insurance was linked with longer adjusted LOS and decreased odds of non-home discharge, compared to insured status. Adequate insurance coverage is therefore paramount in reducing FT risk, as well as racial disparities, in patients undergoing major elective cardiac operations. Culturally-informed strategies to improve financial reimbursement and address systemic inequity are necessary to ensure adequate quality of life in this population.

## Supporting information

S1 Table International classification of disease, tenth revision (ICD-10) codes for identifying study population. (DOCX)

S2 Table Shape and scale parameters of the gamma distributions for each National Inpatient Sample-defined income quartile. (DOCX)

S1 Fig Gamma distributions based on 2019 GINI coefficient. (TIF)
