# Supplementary material for: Socioeconomic disparities in risk of financial toxicity following elective cardiac operations in the United States
Source: PLoS One. 2024 Jan 31;19(1):e0292210. doi: 10.1371/journal.pone.0292210 (PMC10830059; doi:10.1371/journal.pone.0292210)
Supplement: S1 Table — (DOCX) [file pone.0292210.s001.docx]

**Supplemental Table 1.** *International Classification of Disease, Tenth Revision* (ICD-10) Codes for Identifying Study Population

| **Procedure** | **ICD-10 Codes** |
| --- | --- |
| Coronary Artery Bypass Graft Surgery, One Vessel | 0210083, 0210088, 0210089, 021008C, 021008F, 021008W, 0210093, 0210098, 0210099, 021009C, 021009F, 021009W, 02100A3, 02100A8, 02100A9, 02100AC, 02100AF, 02100AW, 02100J3, 02100J8, 02100J9, 02100JC, 02100JF, 02100JW, 02100K3, 02100K8, 02100K9, 02100KC, 02100KF, 02100KW, 02100Z3, 02100Z8, 02100Z9, 02100ZC, 02100ZF, 02100ZW |
| Coronary Artery Bypass Graft Surgery, Two Vessels | 0211083, 0211088, 0211089, 021108C, 021108F, 021108W, 0211093, 0211098, 0211099, 021109C, 021109F, 021109W, 02110A3, 02110A8, 02110A9, 02110AC, 02110AF, 02110AW, 02110J3, 02110J8, 02110J9, 02110JC, 02110JF, 02110JW, 02110K3, 02110K8, 02110K9, 02110KC, 02110KF, 02110KW, 02110Z3, 02110Z8, 02110Z9, 02110ZC, 02110ZF, 02110ZW |
| Coronary Artery Bypass Graft Surgery, Three Vessels | 0212083, 0212088, 0212089, 021208C, 021208F, 021208W, 0212093, 0212098, 0212099, 021209C, 021209F, 021209W, 02120A3, 02120A8, 02120A9, 02120AC, 02120AF, 02120AW, 02120J3, 02120J8, 02120J9, 02120JC, 02120JF, 02120JW, 02120K3, 02120K8, 02120K9, 02120KC, 02120KF, 02120KW, 02120Z3, 02120Z8, 02120Z9, 02120ZC, 02120ZF, 02120ZW |
| Coronary Artery Bypass Graft Surgery, Four or More Vessels | 0213083, 0213088, 0213089, 021308C, 021308F, 021308W, 0213093, 0213098, 0213099, 021309C, 021309F, 021309W, 02130A3, 02130A8, 02130A9, 02130AC, 02130AF, 02130AW, 02130J3, 02130J8, 02130J9, 02130JC, 02130JF, 02130JW, 02130K3, 02130K8, 02130K9, 02130KC, 02130KF, 02130KW, 02130Z3, 02130Z8, 02130Z9, 02130ZC, 02130ZF, 02130ZW |
| Aortic Valve Replacement or Repair | 02RF07Z, 02RF08Z, 02RF0JZ, 02RF0KZ, 02RF47Z, 02RF48Z, 02RF4JZ, 02RF4KZ, 02QF0JZ, 02QF0ZZ, 02QF4JZ, 02QF4ZZ, 02UF07J, 02UF07Z, 02UF08J, 02UF08Z, 02UF0JJ, 02UF0JZ, 02UF0KJ, 02UF0KZ, 02UF47J, 02UF47Z, 02UF48J, 02UF48Z, 02UF4JJ, 02UF4JZ, 02UF4KJ, 02UF4KZ |
| Mitral Valve Replacement or Repair | 02RG07Z, 02RG08Z, 02RG0JZ, 02RG0KZ, 02RG47Z, 02RG48Z, 02RG4JZ, 02RG4KZ, 02QG0JZ, 02QG0ZZ, 02QG4JZ, 02QG4ZZ, 02UG07J, 02UG07Z, 02UG08J, 02UG08Z, 02UG0JJ, 02UG0JZ, 02UG0KJ, 02UG0KZ, 02UG47J, 02UG47Z, 02UG48J, 02UG48Z, 02UG4JJ, 02UG4JZ, 02UG4KJ, 02UG4KZ |
| Tricuspid Valve Replacement or Repair | 02RJ07Z, 02RJ08Z, 02RJ0JZ, 02RJ0KZ, 02RJ47Z, 02RJ48Z, 02RJ4JZ, 02RJ4KZ, 02QJ0JZ, 02QJ0ZZ, 02QJ4JZ, 02QJ4ZZ, 02UJ07J, 02UJ07Z, 02UJ08J, 02UJ08Z, 02UJ0JJ, 02UJ0JZ, 02UJ0KJ, 02UJ0KZ, 02UJ47J, 02UJ47Z, 02UJ48J, 02UJ48Z, 02UJ4JJ, 02UJ4JZ, 02UJ4KJ, 02UJ4KZ |
| Pulmonic Valve Replacement or Repair | 02RH07Z, 02RH08Z, 02RH0JZ, 02RH0KZ, 02RH47Z, 02RH48Z, 02RH4JZ, 02RH4KZ, 02QH0JZ, 02QH0ZZ, 02QH4JZ, 02QH4ZZ, 02UH07J, 02UH07Z, 02UH08J, 02UH08Z, 02UH0JJ, 02UH0JZ, 02UH0KJ, 02UH0KZ, 02UH47J, 02UH47Z, 02UH48J, 02UH48Z, 02UH4JJ, 02UH4JZ, 02UH4KJ, 02UH4KZ |
