# Supplementary material for: Socioeconomic disparities in risk of financial toxicity following elective cardiac operations in the United States
Source: PLoS One. 2024 Jan 31;19(1):e0292210. doi: 10.1371/journal.pone.0292210 (PMC10830059; doi:10.1371/journal.pone.0292210)
Supplement: S2 Table — (DOCX) [file pone.0292210.s002.docx]

**Supplementary Table 2.** Shape and scale parameters of the gamma distributions for each National Inpatient Sample-defined income quartile.

| **Income Quartile** | **Estimated Mean Income ($)** | **Shape Parameter** | | **Scale Parameter** |  |
| --- | --- | --- | --- | --- | --- |
| Lowest | 45,249 | 1.568 | 28,858 | | |
| 2^nd^ | 57,499 | 1.568 | 36,670 | | |
| 3^rd^ | 76,499 | 1.568 | 48,788 | | |
| Highest | 113,784 | 1.568 | 72,566 | | |
