# Supplementary figures and images for: Socioeconomic disparities in risk of financial toxicity following elective cardiac operations in the United States
Source: PLoS One. 2024 Jan 31;19(1):e0292210. doi: 10.1371/journal.pone.0292210 (PMC10830059; doi:10.1371/journal.pone.0292210)

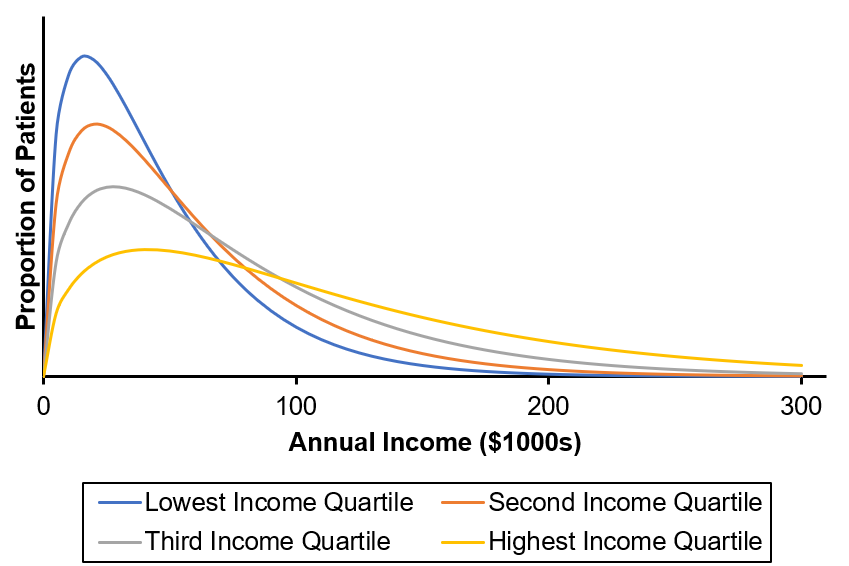

Supplement: S1 Fig — (TIF) [file pone.0292210.s003.tif]
